# A Signature in HIV-1 Envelope Leader Peptide Associated with Transition from Acute to Chronic Infection Impacts Envelope Processing and Infectivity

**DOI:** 10.1371/journal.pone.0023673

**Published:** 2011-08-18

**Authors:** Mohammed Asmal, Ina Hellmann, Weimin Liu, Brandon F. Keele, Alan S. Perelson, Tanmoy Bhattacharya, S. Gnanakaran, Marcus Daniels, Barton F. Haynes, Bette T. Korber, Beatrice H. Hahn, George M. Shaw, Norman L. Letvin

**Affiliations:** 1 Division of Viral Pathogenesis, Beth Israel Deaconess Medical Center, Harvard Medical School, Boston, Massachusetts, United States of America; 2 Department of Medicine, University of Alabama at Birmingham, Birmingham, Alabama, United States of America; 3 Theoretical Division, Los Alamos National Laboratory, Los Alamos, New Mexico, United States of America; 4 Santa Fe Institute, Santa Fe, New Mexico, United States of America; 5 Duke Human Vaccine Institute, Duke University Medical Center, Durham, North Carolina, United States of America; Institut Pasteur, France

## Abstract

Mucosal transmission of the human immunodeficiency virus (HIV) results in a bottleneck in viral genetic diversity. Gnanakaran and colleagues used a computational strategy to identify signature amino acids at particular positions in Envelope that were associated either with transmitted sequences sampled very early in infection, or sequences sampled during chronic infection. Among the strongest signatures observed was an enrichment for the stable presence of histidine at position 12 at transmission and in early infection, and a recurrent loss of histidine at position 12 in chronic infection. This amino acid lies within the leader peptide of Envelope, a region of the protein that has been shown to influence envelope glycoprotein expression and virion infectivity. We show a strong association between a positively charged amino acid like histidine at position 12 in transmitted/founder viruses with more efficient trafficking of the nascent envelope polypeptide to the endoplasmic reticulum and higher steady-state glycoprotein expression compared to viruses that have a non-basic position 12 residue, a substitution that was enriched among viruses sampled from chronically infected individuals. When expressed in the context of other viral proteins, transmitted envelopes with a basic amino acid position 12 were incorporated at higher density into the virus and exhibited higher infectious titers than did non-signature envelopes. These results support the potential utility of using a computational approach to examine large viral sequence data sets for functional signatures and indicate the importance of Envelope expression levels for efficient HIV transmission.

## Introduction

Human immunodeficiency virus (HIV) in acutely infected individuals is markedly less diverse genetically than in chronically infected individuals [1] [2], [3], [4], [5], [6] [7]. Despite the fact that HIV exists in chronic infection as a swarm of genetically related but distinct viruses, called a quasispecies, approximately 80% of new heterosexually transmitted HIV infections are established by a single genetic variant of the virus [8], [9]. The selection of the transmitted founder virus is known to antedate the evolution of selective pressure from an adaptive immune response [10], and likely reflects constraints imposed upon the virus either during transmission or early expansion.

Determining how one or a few of the many viral sequences from the infecting individual successfully establish infection in the new host may elucidate crucial events that occur during mucosal transmission of HIV. Two general mechanisms for this genetic bottleneck have been suggested: either it is the result of a very low probability stochastic event whereby on average only a single virus slips through in a random fashion, or there is active selection for viral variants with specific biological properties, excluding the vast majority of quasi-species. In the two largest studies of sequences from acutely transmitted virus to date, the proportion of individuals infected by a single genetic variant in comparison to multiple variants did not conform to a Poisson distribution. The authors concluded from this finding that genetic constriction at transmission was not likely due simply to a very low probability stochastic event [9], but that active processes were required to produce the observed distribution of multiple versus single virus transmissions [9].

If genetic constriction at transmission results from the active selection of specific viral amino acid sequences, early stages in viral transmission and expansion must favor these selected sequences for interactions with specific extracellular receptors or intracellular co-factors. In fact, this has been shown to be true, as there is a strong preference for transmission of CCR5 tropic over CXCR4 tropic strains of virus [11] [12], [13]. A likely candidate HIV protein to harbor such signatures would be the viral envelope, the initial contact point between the virus and both target cells and the extracellular milieu.

Previous investigation of small sample sizes of early HIV envelopes has failed to detect conclusive commonalities in mutational patterns between transmitted envelopes from different patients [4], [11], [14], although more recent studies have shown that viruses with shorter loop lengths and few potential N linked glycosylation sites are enriched among transmitted viruses [15]. A comparison of envelope sequences of acutely infected individuals and chronically infected individuals was recently completed based on a much larger sample size. Consensus envelope amino acid sequences from forty-three acutely infected individuals were compared to forty-eight consensus sequences from chronically infected individuals, using previously described phylogenetically controlled methods [16]. A hold out set of comparable size was reserved to validate signatures defined in the original data (Gnanakaran, et al., manuscript under review).

Potential signatures were identified at or near the CCR5 co-receptor binding site and the CD4 binding site, as well as at amino acid positions 413–415, where transmitted viruses exhibited loss of a potential N-linked glycosylation site that has previously been associated with escape from broadly neutralizing antibodies. The amino acid position that showed the most dramatic and statistically significant difference between acute/early and chronic envelopes in both the initial and validation analysis was amino acid position 12 of the envelope glycoprotein. Position 12 is variable; within the B clade as well as most other clades, a histidine is the most common amino acid at this position (Gnanakaran, et al, manuscript under review). The amino acid residue at this position was statistically more likely to be stably preserved as histidine in envelopes of acutely transmitted viruses, and was more likely to acquire a different amino acid than histidine in envelopes of viruses from chronically infected individuals. The recurrent pattern of mutation away from histidine during the course of infection suggests that it may be commonly selected against as infections progress. The high frequency of histidine among acute and early viruses, however, demonstrates that viruses carrying the signature histidine are fit and readily transmitted, and the relative absence of the other forms may indicate selection at transmission. Position 12 in the envelope amino acid sequence lies within the leader peptide of the protein. The discovery of an envelope signature at this site suggests a novel role for the leader peptide in regulating envelope characteristics that impact on early infection.

The envelope leader peptide is primarily responsible for directing transport of the nascent polypeptide to the endoplasmic reticulum (ER). Unlike the leaders of other secreted and membrane-bound proteins, it is cleaved post-translationally instead of co-translationally, and this late cleavage has been hypothesized to confer an unusual role for the leader in regulating higher order processing of the envelope protein. The envelope leader has been implicated in the timing of binding of envelope to various ER chaperones responsible for promoting proper folding and glycosylation of envelope, including calnexin and CD4 [17]–[18] [19], [20]. Substitution of the native envelope leader peptide with certain heterologous leaders augmented expression and secretion of envelope [21], while substitution of the leader peptide of a heterologous protein with the envelope leader peptide slowed ER processing of that protein by delaying folding and maturation of glycosylation [17]. HIV envelope synthesized through heterologous leader peptides in the context of a complete provirus resulted in decreased envelope incorporation into viral particles and diminished *in vitro* infectivity [22]. These data suggested that the signal peptide is not merely a passive trafficking signal, but rather an evolving, active modulator of envelope function.

The present studies were initiated to examine the function of the signal peptide signature derived computationally by Gnanakaran and colleagues [manuscript appended]. We hypothesized that a signature located in the leader peptide would manifest itself during envelope synthesis as the leader is cleaved early in the biosynthesis of the glycoprotein. We utilized HIV envelopes from acutely infected individuals to examine the effects of polymorphisms at position 12 on translation of envelope, leader peptide function and viral infectivity.

## Materials and Methods

### Plasmid Constructs and PCR mutagenesis

Transmitted founder *rev-vpu-env* cassettes derived by single genome amplification and cloned into pcDNA3.1/V5-His (Invitrogen) have been described [8]. Position 12 point mutants were generated using two-step PCR. The following mutagenic primers were utilized to introduce single base pair changes at the position 12 site converting histidine or arginine into glutamine (AA01–AA03) or converting non-histidine residue to histidine (AC01–AC03):

AA01: 5′ CCTATGGCAGGAAGAAGCGG

3′ CATCTTATAGCAAAGCCCTTTC

HΔQ_F: GAGGAATTGGCAGCA***A***TTGTGGAAATGG

HΔQ_R: CCATTTCCACAATTGCTGCCAATTCCTC

AA02: 5′ CCTATGGCAGGAAGAAGTGG

3′ CTTATAACAAAGCCCTTTCG

RΔQ_F: GAAGAATTGTCAGC***AA***TTGTGGAGATGGG

RΔQ_R: CCCATCTCCACAATTGCTGACAATTCTTC

AA03: 5′ CCTATGGCAGGAAGAAGCGGAG

3′ CTTATAGCAAAGCCCTTTCC

RΔQ_F: GAGGAATTGTCAGC***AA***TTGTGGACATGGG

RΔQ_R: CCCATGTCCACAATTGCTGACAATTCCTC

AC01: 5′ CCTTAGGCATCTCCTATGGCAGG

3′ GTTATAGAAGAGCCCTTTCTAAGCC

QΔH_F: GAATTGTCAGCACCACTTA

QΔH_R: CCATAAGTGGTGCTGACAATTC

AC02: 5′ CCTATGGCAGGAAGAAACGGAGAC

3′ CATCTTATAGCAAAGCCCTTTC

(gap)ΔH_F: GATCAGGAAGAATTACCAGCACTGGTGGAAATGGGGCAC

(gap)ΔH_R: GTGCCCCATTTCCACCAGTGCTGGTAATTCTTCCTGATC

AC03: 5′ CCTATGGCAGGAAGAAGCGGAG

3′ CATCTTATAGCAAAGCCCTTTC

QΔH_F: GAAGAATTATCAGCACTGGTGGAGAGG

QΔH_R: CCTCTCCACCAGTGCTGATAATTCTTC

For amplification, thermocycler conditions were 95 C for 4 minutes; 30 cycles of 95 C for 30″, 50 C for 30″, 72 C for 4′; 72 C for 20′. In the first PCR step, the 5′ (1) and 3′ (2) mutated fragments were individually generated in two separate reactions using either (1) the 5′ primer starting at the Rev start codon and the reverse internal mutagenic primer, or (2) the forward internal mutagenic primer and 3′ Env primer, respectively. These products were agarose gel purified, combined and introduced into a second stage PCR with the 5′ Rev and 3′ Env primers. Products of the second stage PCR were cloned into pcDNA3.1 by TA ligation.

V5-epitope tagged versions of transmitted envelopes were generated by PCR using the 5′ primers above and the following 3′ primers to eliminate the stop codon of Env:

AA01: CAACAAAGCTCTTTCCAAGCCCTG

AA02: CAACAAAGCCCTTTCGAAGCCCTG

AA03: GAGCAAAGCCCTTTCCAAGCCCTG

AC01: GAGAAGAGCCCTTTCTAAGCCCTG

AC02: GAGCAAAGCCCTTTCAAAGCCCTG

AC03: GAGCAAAGCCCTTTCCAAGCCCTG

These PCR products were cloned in pcDNA3.1. All mutants were verified by sequencing.

### Quantification of Envelope Synthesis

293T cells obtained from the American Tissue Type Collection were maintained in Dulbecco's Modified Eagle Medium with 10% fetal calf serum. Jurkat human leukemia T cell line were also obtained from ATCC and maintained in RPMI supplemented with 10% fetal calf serum. All cells were maintained in a humidified incubator at 37 C with 5% carbon dioxide.

For Jurkat transfections, 2×10^6^ Jurkat cells were transfected with 4 micrograms of plasmid DNA using DMRIE-C (Invitrogen) per manufacturer's protocols, in serum-free, antibiotics-free medium. 10% fetal calf serum was added after 4 hours. Cells were lysed after 48 hour incubation in 1% NP40, 20 mM Tris pH 8.0, 137 mM NaCl, 10% glycerol, 2 mM EDTA with protease inhibitor cocktail (Sigma). Lysates were subjected to SDS-PAGE using the NuPage system (Invitrogen) on a Bis-Tris 4–12% gel run at 200 mV for 1 hour. Transfers were done using the same system to a 0.45 micron nitrocellulose membrane. Blots were probed with 3B3, a mouse monoclonal anti-gp120 antibody (gift of B. Haynes), or anti-V5 antibody (Invitrogen) to assess for envelope. Antibodies to B-actin (Santa Cruz sc-81178) and p24 (Santa Cruz sc-69658) were used as controls.

All experiments were repeated multiple times and representative images are shown.

The Quantity One analysis software (Bio-Rad) was used to quantify relative band intensity on Western blot images.

### P24 ELISA

An HIV-1 p24 enzyme-linked immunosorbent assay kit (Zeptometrix) was utilized to quantify p24 in pseudovirion supernatants. Supernatants were diluted 1∶250 to 1∶5000 in protocol lysis buffer, and incubated overnight on pre-coated plates. Plates were processed per manufacturer's protocols, and samples were read on a Spectramax 384 Plus at 450 nanometers.

### TZM-bl Assay

Pseudovirion supernatants were serially diluted five-fold in 96-well plates in duplicate or triplicate. 2×10^4^ TZM-bl cells (gift of M. Seaman) along with 10 ug/mL of DEAE dextran were added to each well. Samples were co-incubated for 48 hours. 100 microliters of Bright-glo reagent (Promega), containing lysis buffer and luciferase substrate, was added to each well and samples were incubated for 2 minutes at room temperature. Samples were analyzed using a Perkin Elmer Victor 3 luminometer.

### Luciferase reporter

We used the Ready-To-Glow Dual Secreted Reporter Assay (Clontech) to generate and assay leader peptide reporters. We used two-step PCR to generate HIV-1 envelope leader peptide luciferase chimeric proteins. The first step consisted of parallel PCR reactions. One reaction included either AA01, AC01 or the AA01 position 12 mutant as template. Primers for the first reaction included as the forward primer Env specific 5′ leader sequence and as reverse primer a fusion sequence composed of internal envelope signal peptide sequence joined to 5′ luciferase sequence. The second reaction utilized the *Metridia longa* luciferase expression plasmid, pMetLuc2-Control Vector, as the template. Upstream primers for the second reaction included the reverse complement of the fusion primers described above; downstream primers were constructed from 3′ sequence for luciferase. Primer sequences were:

AA01: 5′ ACCGGTGCAATGAGAGTGAAG

Internal_F GATCTGTAGTGCTAAGAGCACCGAGTTC

Internal_R GAACTCGGTGCTCTTAGCACTACAGATC

AC01: 5′ ACCGGTGCAATGAGAGTGAAGG

Internal_F GATTTGTAGTGCTAAGAGCACCGAGTTC

Internal_R GAACTCGGTGCTCTTAGCACTACAAATC

Luciferase: 3′ CGCGGCCGCTCATCACCTGTC

For the first stage of amplification, thermocycler conditions were 95 C for 4 minutes; 30 cycles of 95 C for 30″, 50 C for 30″, 72 C for 60″; 72 C for 10′. For second stage PCR, 1/20^th^ of the volume from the leader peptide and the luciferase PCR reactions were combined with appropriate envelope specific upstream and luciferase specific downstream primers. For the second stage of amplification, thermocycler conditions were 95 C for 4 minutes; 30 cycles of 95 C for 30″, 50 C for 30″, 72 C for 3′ 30″; 72 C for 10′. Products of the second reaction were agarose gel purified and cloned into pcDNA3.1.

10^6^ Jurkat cells were transfected in triplicate with three micrograms of plasmid DNA of the various leader peptide- luciferase chimeras using DMRIE-C reagent. Cells were co-transfected with one microgram of plasmid DNA for pSEAP2-Control Vector, expressing secreted alkaline phosphatase from its native leader peptide.

150 microliters of each of the triplicate supernatants was harvested at 12, 24, 36, 48 and 60 hours post transfection, without disrupting the underlying cells. Supernatants were analyzed separately for luciferase and SEAP activity per manufacturer's instructions. Chemiluminescence was measured on a Perkin Elmer Victor 3 luminometer.

### Pseudovirion generation and purification

293T cells were plated to reach 90 – 95% density in T-75 flasks by day of transfection. Either 16 micrograms of SG3ΔEnv (AIDS Reagent Program) and 4 micrograms of pcDNA3.1 envelope (four to one ratio) or 10 micrograms (one to one ratio) of each construct was transfected using lipofectamine 2000. Supernatants were harvested 24 – 48 hours later and 0.2 micron filtered. These were used either directly in the TZM-bl assay or subsequently clarified by centrifugation at 4,000 RPM for 30 minutes followed by ultracentrifugation over 20% sucrose w/v in phosphate buffered saline at 27,000 RPM for 2 hours in an SW-28 swinging bucket rotor (Sorvall). Supernatants were decanted and pellets dried briefly before resuspension in 50 – 100 microliters of PBS.

For quantitative Western blot, five T-75 flasks each for pseudovirions of AA01 and AC01 were pooled prior to ultracentrifugation. Pseudovirions were purified as above. Two-fold serial diluted purified ConSΔCFI140 (from Dr. Huaxin Liao) and HIV-1 p24 protein (Protein Sciences Corp., CT) and of pseudovirion samples AA01 and AC01 were resolved by SDS-PAGE on 4–20% tris-glycine gels. Following electrophoresis, proteins were transferred to polyvinylidene difluoride (PVDF) membranes, incubated with PBS-T with 5% non-fat milk for 1 hour at room temperature, and then incubated with goat-anti-gp120 antibody (USbiological, Swampscott, MA) or rabbit anti-p24 antibody (NIH reagent program) overnight at 4°C. Protein-bound antibody was probed with rabbit-anti-goat IgG-HRP or goat anti-rabbit IgG-HRP (Southern Biotech, Birmingham, AL) and developed using an enhanced chemiluminescence (ECL) detection system (GE Healthcare, Piscataway, NJ), respectively. The Env and gag contents were measured using the VersaDoc 4000 Imaging System (Bio-Rad, Hercules, CA). The density of each band from the pseudovirion samples was interpolated into a standard curve derived from a linear regression of density values from serial dilutions of purified ConSΔCFI140 or HIV-1 p24 protein.

### Mathematical model

We utilized a previously published model of acute infection studied by Nowak et al. for SIV and Stafford et al. for HIV [23], [24]. The equations in the model relate three variables: *V*, viral load; *T*, activated, uninfected CD4+ T cells; T*, infected CD4+ T cells. Activated, uninfected target cells are presumed to be renewed at a constant rate, λ, and die at a rate of *d* per cell. Infected cells die at a rate of δ, and plasma virus decays at a rate of *c*. New virions are produced at a rate of π per infected cell. Uninfected cells are infected at a rate proportional to the amount of free virus, governed by the proportionality constant *k*, the infectivity.
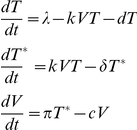
Equations for the exponential viral growth rate, *r_0_*, and the steady-state viral load, *Vss*, were taken from Nowak, et al. [23]. The free on-line software Math Mechanixs was used to graph equations.

## Results

### Position 12 lies within a charged domain of the leader peptide

Leader peptide sequences of most secreted or membrane-bound proteins are characterized by a positively charged amino terminus, a short hydrophobic span and a carboxy terminus that culminates in a cleavage site. The HIV envelope leader peptide, at thirty amino acids, is longer than most leader peptides and more highly positively charged, with on average five basic amino acids [21]. The proposed HIV envelope signature at residue 12 lies within the positively charged N-terminus of the leader sequence. Consistent with this, sixty-nine of seventy-nine transmitted envelopes analyzed from the CHAVI 001 cohort contained histidine at this position. Four of these seventy-nine leader peptides also had arginine at this residue. We hypothesized that because arginine at position 12 would preserve the overall positive charge of this region of the leader, it would maintain the function of a histidine signature. In contrast, fifty-seven out of the one hundred and eleven analyzed chronic envelopes had an uncharged glutamine or proline, or eliminated the charged residue altogether from position 12, leading to a change in the charge distribution of the typically basic N-terminus of the leader. We hypothesized that non-basic residues at position 12 found in chronic envelopes represent evolution away from the transmission genotype.

Because of the leader peptide's primary role in trafficking of newly translated proteins, variation at position 12 of HIV envelope might be expected to manifest itself phenotypically at the level of protein synthesis. Alterations in the rate of endoplasmic reticulum transport of gp160 early during HIV infection may result in higher throughput envelope synthesis, potentially altering the rate of virion production or the protein content of virions produced. Alternatively, the envelope leader's unusual delayed cleavage and putative role in modulating gp160 interactions with calnexin and other endoplasmic reticulum chaperones suggested a role for leader peptide polymorphisms in modulating envelope structure and glycosylation [25], [26], [27], potentially impacting on downstream envelope-host interactions.

In order to study the biochemistry of the leader peptide with the position 12 signature, we obtained functional *rev-vpu-env* cassettes from 14 acutely infected individuals as previously described [8]. These cassettes contained the *env* genes of transmitted/founder HIV-1 cloned into pcDNA3.1 (Invitrogen) and contained the entire gp160 open reading frame, including 5′ sequence extending to the Rev start codon [8]. A portion of the amino acid alignments of the leader peptides of these sequences are shown in Table 1. Complete nucleotide sequences for these envelopes are accessible through GenBank (EU574937–EU579293) [8], as are all transmitted and chronic envelopes used in the initial analysis that defined the position 12 signature (Gnanakaran, et al, manuscript under review). Characteristics of the study subjects at the time at which viruses were isolated, including patient viral load and Fiebig stage [28] are detailed in Table S1 and in reference [8].

**Table 1:** Leader peptide sequences of transmitted envelopes.

Specimen Identifier												12			Pseudo
*TT31P.2F10.2792*	M	R	V	K	E	T	K	R	N	W	Q	**H**	L	-	*AA01*
*BORId9.4D7.1410*	M	R	A	K	E	I	R	K	N	C	Q	**R**	L	-	*AA02*
*TT27P.8H1.2730*	M	R	A	K	E	I	R	K	N	C	Q	**R**	L	-	*AA03*
*700010040.C9.4520*	M	R	V	M	G	I	R	K	N	Y	Q	**H**	L	-	*AA04*
*PRB931_06.TC3.4930*	M	R	V	M	G	I	R	K	N	Y	Q	**H**	L	-	*AA05*
*12008.08_B33_4830*	M	R	V	M	E	I	R	R	N	Y	Q	**H**	W	-	*AA06*
*9021_14.B2.4567*	M	R	V	K	G	I	R	K	N	C	Q	**Q**	H	L	*AC01*
*9020.20 A13*	M	R	V	K	G	I	R	K	N	Y	-	**-**	W	-	*AC02*
*PRB956.04B20*	M	R	A	T	G	M	R	K	N	Y	Q	**Q**	W	-	*AC03*
*1059_09.A4.1460*	M	R	V	T	E	I	R	K	N	Y	-	**-**	L	-	*AC04*
*SC33_4A4_2589*	M	R	V	K	G	I	R	R	N	W	Q	**G**	L	-	*AC05*
*SC33_4H1_2589*	M	R	V	K	G	I	R	R	N	W	Q	**G**	L	-	*AC06*
*700010058_A4_4375*	M	R	V	T	G	I	K	K	N	Y	Q	**N**	L	-	*AC07*
*SUMA_2*	M	K	V	K	G	I	R	K	N	Y	-	**-**	F	-	*AC08*

Alignment of first 14 amino acids of envelope for 14 transmitted clones, grouped by position 12 signature (bolded). Each sequence has a unique specimen identifier as well as a pseudonym, by which it will be referred in this paper.

**Figure 1:**
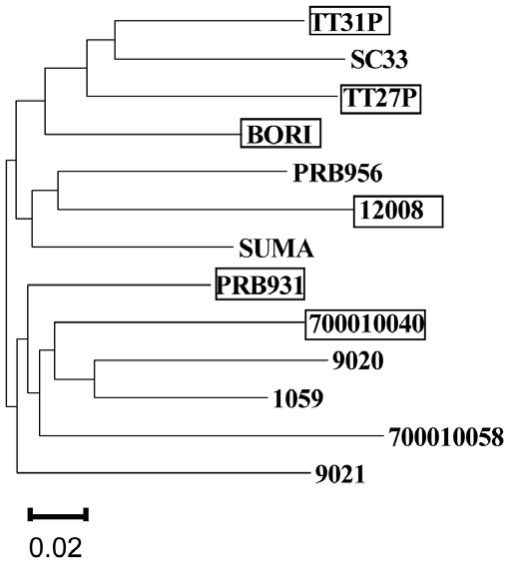
Maximum likelihood phylogenetic tree for envelope sequences used in this study. Sequence alignments and ML tree were generated using Seaview 4.0 [49]. Envelopes with the basic position 12 residue are boxed and those lacking the signature are not boxed.

Six of these envelopes were selected at random from the entire cohort because their leader peptide sequences contained a position 12 signature histidine or a similarly basic arginine at position 12. The remaining eight envelopes were specifically chosen because they are atypical in the transmitted envelope cohort in that they contain a variety of non-canonical residues at this position, including glutamine, glycine, asparagine, or a gap in the alignment. There was no phylogenetic clustering of envelopes bearing the signature distinct from those lacking the signature (Figure 1), suggesting that the phenotypic similarities among signature containing envelopes could not be explained by a shared evolutionary history.

### Non-signature envelopes exhibit lower steady-state expression

**Figure 2:**
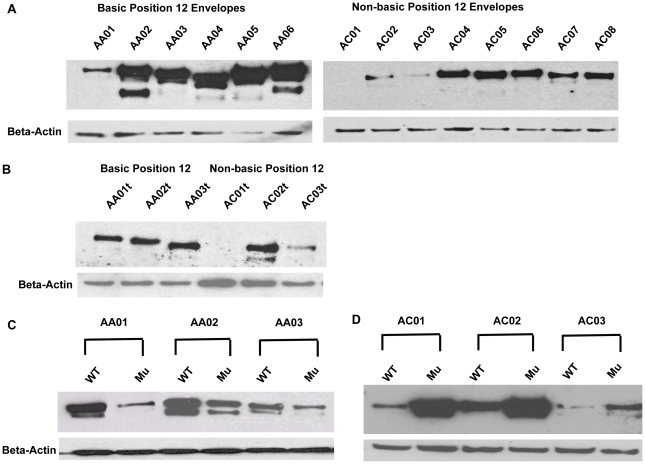
Position 12 signature enhances steady-state envelope expression in Jurkat cells. (A) Transmitted envelopes were expressed in Jurkat cells by transient transfection and cell lysates analyzed by SDS-PAGE and Western blot, probed with the 3B3 monoclonal anti-gp120 antibody. Equal well loading was confirmed by subsequent membrane probing with a monoclonal antibody to beta-actin. Envelope signal intensities were quantified by Quantity One (BioRad). Mean signal for AA01–AA06: 3433 arbitrary units, standard deviation: 973. Mean and standard deviation for AC01–AC08: 1379 and 481, respectively. P value by Students' two-tailed T-test = .0002 for difference between the two groups. (B) Six transmitted envelopes were carboxy-terminus V5 epitope tagged and expressed in Jurkat cells. Western blots were probed with a monoclonal antibody to V5. (C) Single point mutations were introduced into the sequences of three envelopes, converting the native histidine or arginine (WT) into non-basic glutamine (Mu). Mean decrement in protein expression caused by position 12 mutation was 61%, with a standard deviation of 16% among the three envelopes analyzed. (D) Single point mutations were introduced into three envelopes converting the native glutamine or alignment gap (WT) into basic histidine (Mu). Mean increase in protein expression caused by position 12 mutation was 3.6 fold with a standard deviation of 1.9 fold.

We began our exploration of the effects of the position 12 polymorphism on HIV biology by examining envelope translation. We transiently transfected Jurkat T cells with the 14 transmitted envelope constructs described previously. Forty-eight hours after transfection, we compared expression by Western blot, (Figure 2A). Comparable transfection efficiencies were confirmed by co-transfection with a GFP expression plasmid (data not shown). We observed higher levels of steady-state envelope expression by envelopes with a basic residue at position 12 (AA01– AA06) in comparison to those lacking the signature (AC01 – AC08). Using band densitometry to compare relative expression levels, we found that, on average, signature envelope expression was 2.5-fold higher than non-signature envelope expression; the difference in signal intensity between the two groups was highly significant (p<.005).

A trivial explanation for the apparent differences in expression between these groups of envelope constructs might be differences in affinity of the anti-gp120 antibody for the different envelope proteins. To control for this, we generated epitope-tagged versions of three signature and three non-signature envelopes utilizing a carboxy-terminus V5 epitope tag encoded within the pcDNA3.1 expression vector. We probed Western blots of transfections of these constructs with an anti-V5 antibody, and again observed a trend to reduced expression in non-signature envelopes (Figure 2B).

The acutely transmitted envelopes described above were all isolated from different patients, and hence have significant sequence heterogeneity throughout the envelope protein. To demonstrate that differences in expression were due specifically to polymorphisms at position 12, we arbitrarily selected three basic residue-bearing envelopes, AA01, AA02 and AA03, and used site-directed PCR mutagenesis to convert the histidine or arginine residue into a glutamine, a relatively common amino acid variant found at this position. We cloned these position 12 envelope mutants into pcDNA3.1, and confirmed the integrity of the full-length envelopes by sequencing. We expressed these position 12 mutant envelopes, and the parent envelopes, in Jurkat cells and observed an over 60% decrement in expression associated with the point mutations (Figure 2C).

We performed the converse experiment using three non-signature envelopes, AC01, AC02 and AC03, and replaced the glutamine or alignment gap at position 12 of these leader peptides with histidine. We observed an approximately three-fold increase in steady-state protein expression of these envelopes after mutation of this residue (Figure 2D), confirming that a basic position 12 residue is important for optimal expression of envelope in lymphocytes.

### Non-signature leader peptide polymorphisms diminish ER targeting

A plausible explanation for the differences in steady-state protein expression among position 12 variants is that position 12 histidine or arginine is critical for the trafficking efficiency of the leader peptide in certain cell types, and loss of this basic residue results in misdirected nascent peptide and loss of fully synthesized envelope. In general, the success of appropriate targeting of secreted or membrane-bound proteins to the endoplasmic reticulum appears to vary significantly between leader peptides, with misdirected proteins locating to the cytoplasm where they are degraded [29], [30]. Furthermore, polymorphisms within endoplasmic reticulum targeting sequences are relevant for disease states: an inherited mutation in the leader peptide sequence of factor VII has been shown to result in mistargeting of the nascent polypeptide to the cytoplasm and a reduction in overall expression of the mature Factor VII protein; this endoplasmic reticulum-trafficking abnormality underlies the heritable coagulation disorder associated with this mutation [31]. We thus investigated whether higher levels of envelope translation among transmitted viruses are driven by increased efficiency of targeting of nascent envelope to the endoplasmic reticulum.

We employed a protein secretion reporter strategy to address the question of whether a position 12 signature bearing leader peptide is a more effective trafficking signal than a non-signature leader. Heterologous reporter proteins, such as secreted alkaline phosphatase, have been used to examine the regulation of secreted protein synthesis, because these reporters can be detected using standardized, rapid assays, without need for Western blot. For our reporter, we decided to use the luciferase of the marine copepod *Metridia longa*, which possesses an endoplasmic reticulum targeting signal sequence [32]. We used fusion PCR to replace the native *Metridia longa* luciferase leader peptide, MDIKVVFTLVFSALVQA, with the envelope leader peptides of (1) AA01; (2) position 12 histidine to glutamine mutant of AA01; (3) AC01.

**Figure 3:**
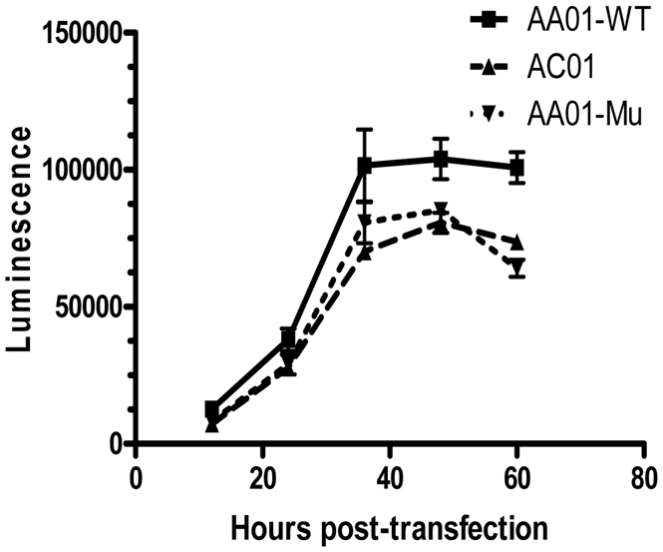
Position 12 polymorphism influences efficiency of leader peptide in regulating protein transport through the secretory pathway. Secreted luciferase reporter constructs were generated to help quantify leader peptide efficiency. Fusion PCR was utilized to substitute the 31 base pair leader peptide of HIV-1 envelope for the 18 base pair Metridia longa secreted luciferase leader peptide. Secreted luciferase constructs were generated bearing the leader peptides of envelopes AA01, AC01 and AA01-Mu containing the histidine to glutamine mutation. These were co-transfected in triplicate into Jurkat cells with a human secreted alkaline phosphatase control vector (pSEAP2, Clontech). Culture supernatants were collected at 12, 24, 48, 36 and 60 hours post-transfection, and analyzed in parallel for luciferase and SEAP activity. Luciferase activity was normalized to the SEAP control, and mean and standard deviation of triplicate transfection results are plotted. Two-tailed Student's T test for comparison of AA01 to AC01 (p = 0.0171) and AA01 to AA01-mu (p = 0.0318) were significant, while comparison of AC01 to AA01-mu revealed no significant difference.

We transfected Jurkat cells with the leader peptide reporter constructs and measured supernatant luciferase activity post-transfection (Figure 3). We co-transfected a secreted alkaline phosphatase (SEAP) reporter plasmid under control of a different promoter and the native SEAP leader peptide, and measured SEAP activity in the supernatant as a control for transfection. We observed detectable luciferase activity for all constructs as early as 12 hours after transfection. This activity was maximal at 36 to 48 hours post-transfection, plateaued and then began to diminish at 60 hours post-transfection. At peak and plateau, the signature bearing envelope, AA01, exhibited 25 – 30% higher luciferase activity than both the non-signature bearing envelope AC01 and the position 12 mutant envelope, AA01-mu. A slightly lesser magnitude difference in luciferase activity was observed at earlier time points. These results suggest that polymorphisms at position 12 directly impact leader peptide efficiency in directing nascent polypeptides to the endoplasmic reticulum, and that this influence on leader function is not specific to HIV-1 envelope but can be imparted to any secreted protein downstream of the leader.

### Non-signature envelopes are less infectious *in vitro*

Because the leader peptide is cleaved early in the synthesis of the HIV Envelope, any phenotype dictated by an HIV leader peptide polymorphism should be manifest prior to or during endoplasmic reticulum processing of gp160. If *in vivo* selection for a signature occurs, there must be physiologic consequences downstream of these biochemical alterations to envelope that are susceptible to selection pressure. A signature imbedded in the leader peptide of envelope could influence the quantity of glycoprotein produced and incorporated and thus virion infectivity.

**Figure 4:**
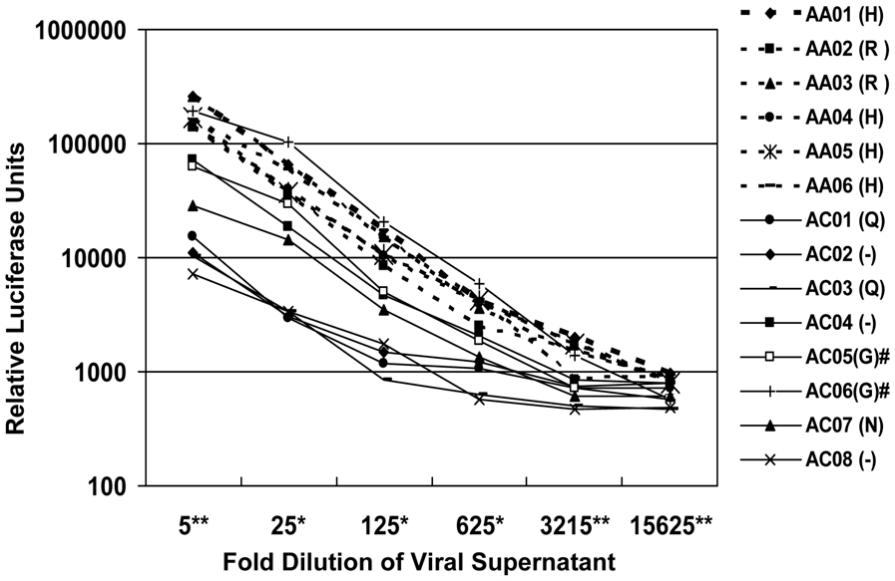
Pseudovirions bearing leader peptide signature envelopes are more infectious in a single round infectivity assay. HIV-1 pseudovirions were generated by co-transfection of 293T cells with pcDNA 3.1 transmitted envelopes and SG3deltaEnv, a plasmid encoding all HIV-1 structural and accessory proteins except for envelope. SG3deltaEnv and the envelope constructs were transfected at a molar ratio of approximately four to one. Culture supernatants were collected 24 to 48 hours after transfection, and passed through a 0.2 micron filter. Five-fold serial dilutions of supernatants were incubated in 96-well plates with 2×104 TZM-bl cells, a reporter cell line expressing HIV-1 co-receptors as well as a Tat-sensitive luciferase reporter. Pseudovirus and TZM-bl cells were incubated for 48 hours before luciferase activity was quantified. This experiment is representative of multiple studies. Six basic (dashed lines) and eight non-basic (solid) transmitted envelopes were assayed; position 12 residue is indicated parenthetically. Relative luciferase activity at each dilution was compared between all basic and non-signature envelopes by Mann-Whitney test. Dilutions at which p-values for comparison are <.05 indicated by *, and <.01 indicated by **.

To determine the impact of position 12 polymorphisms on virion production and infectivity, we developed an *in vitro* complementation assay. By pseudotyping the transmitted envelopes with a uniform set of other viral proteins, we were able to evaluate the effects of envelope polymorphisms on virus infectivity in isolation of other variables. We generated pseudovirus by co-transfection of the envelope constructs with the SG3ΔEnv packaging vector, at a four to one ratio of packaging vector to Envelope. This packaging vector expresses all non-Envelope structural and accessory proteins. We normalized harvested supernatants by p24 ELISA and applied the supernatants to a standard reporter cell line—the TZM-bl HeLa cell line that has been stably modified to express CD4, CXCR4 and CCR5, and which contains a luciferase reporter driven by an HIV-1 LTR promoter [33]. We measured luciferase activity as a proxy for single round infectivity of these non-replicating viruses. Relative luminescence was plotted as a function of the dilution of the viral supernatant added to the reporter cells (Figure 4).

The results showed that envelopes bearing the position 12 signature were generally considerably more infectious than envelopes with non-signature position 12 residues, the only exception being AC06 which encoded a glycine at position 12, yet appeared to generate the most infectious pseudovirions.

Pseudoviruses constructed using Envelopes with glutamine or a gap in the alignment were the least infectious in this assay. We compared luminescence of all of the histidine or arginine containing enveloped pseudoviruses to that of the non-signature enveloped pseudoviruses using the Mann-Whitney test; at all dilutions of virus, the basic position 12 enveloped viruses exhibited significantly greater infectivity than non-signature enveloped viruses. These results indicate that the presence of histidine or arginine residues at position 12 of a transmitted envelope is correlated with greater *in vitro* pseudovirus infectivity than most other common polymorphisms at this site. Interestingly, envelope AC05, which has a glycine at position 12, was comparably infectious to position 12 signature envelopes, suggesting that there are likely other determinants of infectivity that can modify and ameliorate the position 12 phenotype.

We next sought to determine if polymorphism at position 12 is alone sufficient to produce differences in Envelope infectivity. We used envelopes containing point mutations at the signature to generate pseudovirions and assayed these in TZM-bl cells. We found that specific mutation at position 12 from histidine to a non-histidine residue did not substantially reduce pseudovirion infectivity. Similarly, mutation of a position 12 non-histidine residue to histidine was insufficient to increase pseudovirion infectivity (data not shown). This suggests that position 12 may not be the sole determinant of the infectivity phenotype we have observed, but may be one component of a larger multi-locus transmission motif.

### Non-signature envelopes are incorporated at lower density into virions

**Figure 5:**
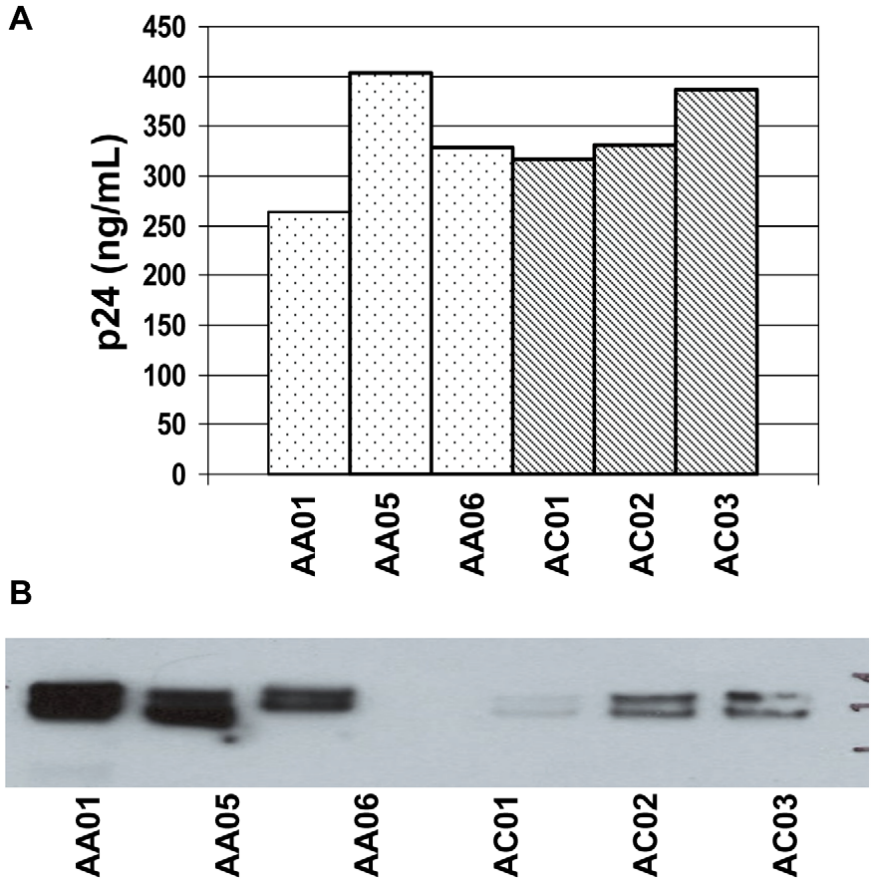
Position 12 signature is associated with increased envelope incorporation into pseudovirions. (A) Pseudovirions were generated in 293T cells, and culture supernatants were layered over 20% sucrose and ultracentrifuged at 27,000 RPM for 2 hours. P24 was quantified by ELISA (Zeptometrix). (B) Pelleted pseudovirions were normalized for p24, and subsequently analyzed by Western blot using the 3B3 antibody.

To examine envelope particle incorporation, we generated pseudovirions with transmitted envelopes and purified these pseudovirions by ultracentrifugation through a 20% sucrose cushion. We quantified p24 in pelleted virus and showed no difference in p24 incorporation associated with the position 12 polymorphism (Figure 5A). We subsequently used Western blot to compare envelope incorporation into pseudovirions and observed that signature-bearing envelopes were incorporated at a higher density into pseudovirions than non-signature-bearing envelopes (Figure 5B). The differences in envelope incorporation into pseudovirions were consistent with the differences in protein synthesis between the signature and non-signature envelopes we had previously observed in Jurkat transfections.

**Figure 6:**
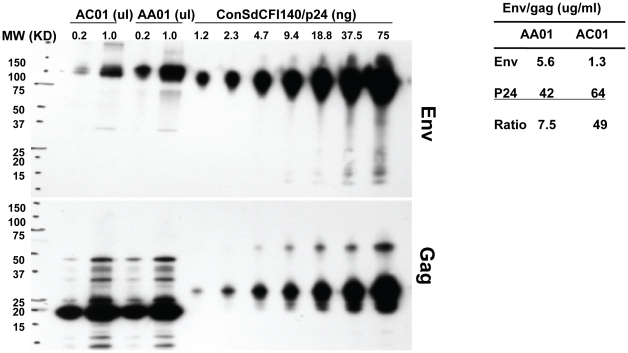
Precise quantification of p24 and envelope content in pseudovirions. Fifty milliliter volumes of 293T pseudovirion supernatants were generated for Envelopes AA01 and AC01. Supernatants were pelleted over a sucrose cushion, and resuspended in PBS. Equal volumes of resuspended AA01 and AC01 were analyzed by SDS-PAGE. Varying dilutions of a previously quantified virus were run simultaneously. Protein signal was measured by densitometry, and p24 and envelope in AA01 and AC01 pseudovirus were quantified by comparison to dilutions of known virus. The ratio of p24 to envelope was calculated for both samples.

We then attempted to quantify more precisely the ratio of Envelope to Gag in the pseudovirions. Large quantities of AA01 and AC01 pseudovirus were prepared and purified, analyzed by Western blot and compared to quantified standards for both Envelope and Gag (Figure 6). For a similar quantity of p24, AA01 pseudovirions contained more than six times as much envelope as AC01 pseudovirions. The ratio of Gag to Envelope in the signature-containing pseudovirion was 7.5∶ 1, much lower that the more commonly reported virion Gag: envelope ratios of 40 – 60∶ 1, whereas this ratio in the non-signature pseudovirus was 49∶1.

**Figure 7:**
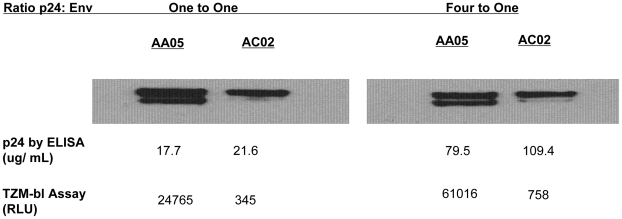
Alteration of ratio of pseudovirion p24:envelope does not alter differential pseudovirion incorporation of envelope between signature and non-signature envelopes. Position 12 histidine bearing envelope AA05 and non-histidine bearing envelope AC02 were co-transfected with SG3deltaEnv at two different ratios of plasmid DNA to generate pseudovirions in 293T cells. Pseudovirions were purified by ultracentrifugation over 20% sucrose. Pellets were resupended and analyzed by Western Blot for envelope content and by p24 ELISA for Gag content. Pelleted pseudovirus was also applied to TZM-bl reporter cells and luciferase activity was measured as an indication of infectivity.

Pseudovirions in these experiments were generated by co-transfection of separate plasmids for envelope and for other structural proteins, at a ratio of four to one, utilizing larger amounts of non-envelope plasmid than envelope plasmid DNA. We queried whether altering the ratio of transfected DNA and consequently the ratio of translated viral envelope and Gag proteins might affect the phenotype of the pseudovirions. We hoped to determine whether increasing the relative quantity of envelope in the transfection might account for the phenotypic differences we observed between signature and non-signature envelopes. We transfected cells with either a 1∶1 or a 4∶1 ratio of SG3deltaEnv to envelope plasmid and purified the pseudovirions. We analyzed similar quantities of pelleted virus for envelope, p24 incorporation and single round infectivity (Figure 7). Envelope incorporation was not altered by modulation of the ratio of transfected DNA. The discrepancy in envelope incorporation between signature and non-signature pseudovirions was similar at both transfection ratios. In contrast, p24 incorporation was reduced by four to five fold at a transfection ratio of 1∶1 in comparison to a transfection ratio of 4∶1. This was true for both signature and non-signature pseudovirions. These results suggest that envelopes with the position 12 signature were incorporated at higher density into virions.

### Computational Model of Infectivity in Early and Late Infection

One intuitively straightforward hypothesis regarding why a signature that associates with infectivity of HIV *in vitro* may be selected for during early infection is that it is important during initial expansion of the virus upon infection, and lost during chronic infection, when other factors may play a stronger selective role. It is possible that during chronic infection a steady-state develops in viral replication and target cell populations. At this steady-state, limitations on target cell numbers and immune susceptibility may play a more important role in viral propagation than do elements that marginally augment viral infectivity. To investigate this hypothesis, we used mathematical models to explore the relationship of viral infectivity to viral load during early and late infection. Similar ideas of trade offs during the life history of a population have been developed in ecology as first proposed by MacArthur and Wilson [34] and are called *r/K* selection theory, where *r* denotes the growth rate of a population and *K* denotes the carrying capacity of the environment.

Mathematical models can be used to evaluate the plausibility of hypotheses about the relationships between different viral and host characteristics and clinical consequences of infection [35], [36]. Previously published models derived by Nowak et al. and by Stafford et al. have been used to approximate the dynamics of *in vivo* SIV and HIV viral load after infection and prior to the exertion of substantial immunologic pressure [23], [24], [37]. These models predict that the initial growth rate of virus should be exponentially dependent on viral infectivity:
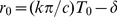
(1)where *r_0_* is the exponential viral growth rate before target cells become rate limiting, and *k* is the viral infectivity. Other important factors that govern the initial growth rate of virus include the number of infectious particles produced per cell (π), the number of target cells at time zero (*T_0_*), and the rates of decay of plasma virus (*c*) and infected cells (δ).

**Figure 8:**
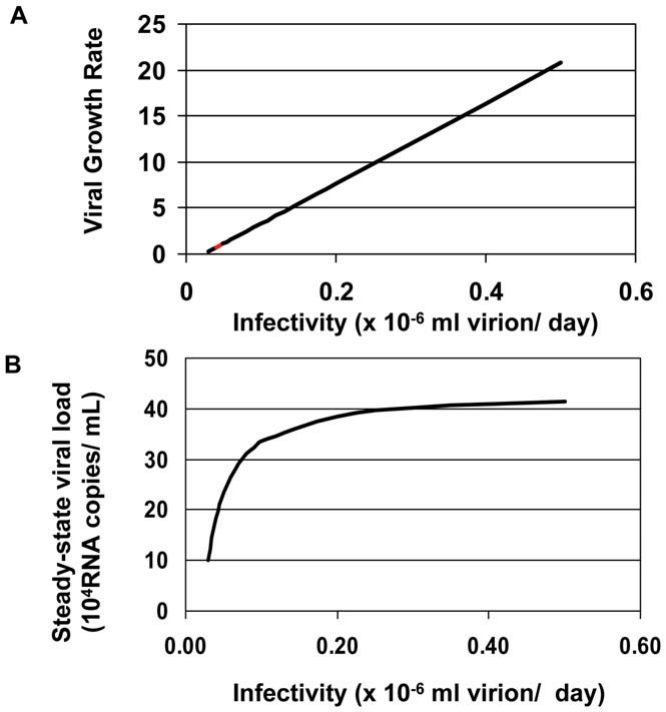
Mathematical modeling of the role of viral infectivity in acute and chronic infection. (A) The exponential growth rate of virus during early infection was plotted as a function of viral infectivity, *k*, using the equation: *r_0_ = (k p/c)T_0_* – δ. Estimates for *p*, *c*, T_0_ and δ were taken from Stafford, et al. [39]. (B) Viral load at steady-state was plotted as a function of viral infectivity, *k*, using the equation: *Vss* = (πλ)/(δ*c*) – *d*/*k*, also taken from Stafford, et al. [39] and Nowak, et al. [40].

We used a previously studied data set of viral loads from ten acutely infected individuals to validate our hypothesis that viral load increases exponentially with viral infectivity [24]. Using equation (1) with empirically derived values for the viral production rate, the viral and infected cell decay rates, and varying the viral infectivity, we demonstrated that as infectivity increases, the exponent governing the rate of viral expansion also increases linearly (Figure 8A). These results suggest that during initial expansion, if target cell availability is not limiting, viral load depends exponentially on viral infectivity. This implies a strong selection pressure early in infection for viruses that have higher levels of infectivity, although it does not necessarily exclude a fundamental role for envelope expression levels in the earliest events of transmission.

During chronic infection, we presumed that virus attained a steady-state with the rates of viral decay and new viral production being in balance. Furthermore, we assume that the total number of target cells remained relatively constant. This is a reasonable first-order approximation for chronic infection during which total CD4+ T cell number does not fluctuate significantly over the short term. Under these approximations, the steady-state viral load, *V_ss_*, becomes [24]:
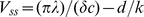
(2)Where λ represents the renewal rate of uninfected target cells, *d* is the death rate of uninfected target cells, and the other variables are as described for equation 1. We again used a data set from acutely HIV-1 infected individuals to plot the change in steady-state viral load as a function of infectivity, *k* (Figure 8B). One can see from equation (2) that as the ratio of the uninfected cell death rate, *d*, to the viral infectivity, *k*, increases, the influence of infectivity on steady-state viral load increases: thus, on the plot, at low values of infectivity, small changes in infectivity, give rise to substantial changes in steady-state viral load. However, as the ratio of *d* to *k* decreases with increasing viral infectivity, the viral steady-state level plateaus. In the data set given in Stafford et al. [24] from which our parameter estimates were derived, the median viral infectivity of the 10 patients studied was 0.65×10^−6^ ml virion^−1^ day^−1^, with a maximum estimate of 4.80×10^−6^ ml virion ^−1^ day^−1^, and minimum estimate of 0.19×10^−6^ ml virion^−1^ day^−1^. These values of infectivity all lie well within the plateau of the curve, implying that, for the range of infectivity observed in acutely infected individuals, changes in viral infectivity should not substantially influence steady-state viral load.

Conceptually, these results suggest that at steady state, target cell populations become limiting, and that under these conditions, the quantity of free virus governs the effectiveness with which the virus is able to access these sparse targets. According to this model, viruses that achieve and sustain higher plasma concentrations are better able to access targets than slower replicating or more quickly cleared viruses. Infectivity, which reflects the ability of a virus to compete for a given target cell, does not impart a significant competitive advantage unless there is very high turnover of uninfected target cells. We hypothesize that this change in the relevance of infectivity during early and chronic phases of infection may be responsible for the importance of the leader peptide signature.

## Discussion

In this study, we offer *in vitro* evidence for the physiologic function of an HIV-1 signature identified through computational analyses of acute and chronic envelope sequences undertaken by Gnanakaran and colleagues. They found histidine to be significantly enriched at the amino acid 12 position of transmitted founder envelopes in comparison to chronic envelopes, where this residue was more likely to mutate to an amino acid other than histidine. We have demonstrated that the presence of a histidine or similarly positively charged arginine at this position, in comparison to non-basic residues, is associated with higher envelope expression and virion incorporation levels, and may influence viral infectivity.

These findings add to the growing literature that leader peptides are not interchangeable shipping labels; rather, they are actively evolving, protein-specific, regulatory elements, and this is reflected in the extreme sequence heterogeneity among different leaders [38]. It has previously been shown that not only do leaders vary in the efficiency with which they target their proteins to the ER, but that some leaders mediate context dependent ER trafficking, directing their messages away from the ER during cellular stress [29], [30], [39]. Specifically, the HIV-1 envelope leader peptide has previously been shown to alter the expression of reporter proteins to which it has been affixed [17]. We used a secreted luciferase reporter with differing envelope leader peptides to show that a change from a basic to a non-basic residue in the positively charged amino-terminus of the leader altered trafficking efficiency. Interestingly, the magnitude of difference in luciferase activity between signature and non-signature leader peptide reporters of 30% (Figure 3) is significantly smaller than the two to three fold difference in full length envelope protein expression we observed by Western blot (Figure 2B). One possible explanation for this difference may be that the position 12 polymorphism has pleiotropic effects on envelope synthesis, influencing not only trafficking of nascent protein to the endoplasmic reticulum, but also affecting the rate of processing within the endoplasmic reticulum. For example, if a polymorphism delays leader peptide cleavage, it might slow processivity through the endoplasmic reticulum, increasing steady-state levels of envelope [17], [40]. A second explanation for the differences in magnitudes observed in our assays may have to do with the nature of the reporter protein used in these studies. Luciferase does not undergo the significant post-translational modification within the ER that envelope glycoproteins do, and has a much shorter transit time from initiation of synthesis to the plasma membrane. The prolonged retention time of envelope within the endoplasmic reticulum may amplify differences in trafficking efficiency. Overall, our findings demonstrating the sensitivity of HIV-1 envelope synthesis to alterations in the leader peptide are consistent with previous studies that have shown that replacement of the native envelope leader peptide with a heterologous leader changes expression and secretion of envelope [21] [41].

We have shown a strong association between the presence of the position 12 polymorphism and viral infectivity. This difference in infectivity correlated with higher levels of signature envelope incorporation into mature pseudovirions. It has previously been shown that higher envelope content results in virions with higher affinity for cellular co-receptors and greater infectivity [41], [42], [43], [44]. Furthermore, tampering with the HIV-1 envelope leader peptide in the context of a complete provirus resulted in alterations in envelope incorporation and changes in virion infectivity [22]. Thus, it is plausible that a position 12 histidine facilitates increased rates of envelope translation, producing virions with higher levels of envelope content that are therefore more infectious.

Interestingly, while we were able to abrogate the envelope translation phenotype by selective mutation of position 12 from basic to non-basic, we were unable to restore the infectivity phenotype by selective mutation of position 12. This suggests that there are other envelope domains that in conjunction with the position 12 signature contribute to the transmission phenotype. Thus, the association between position 12 and infectivity may reflect an association between the signature and other transmission-associated residues throughout envelope. This hypothesis is consistent with our observation that the differences in *in vitro* infectivity between signature and non-signature viruses are more dramatic than are either translation or envelope incorporation differences; there may be more than one mechanism modulating the infectivity phenotype. Position 12 may be one component of a larger transmission motif comprised of non-contiguous polymorphisms at multiple sites. In order to identify other residues in the transmission motif, one would need to probe the combined effects of polymorphisms at position 12 with other signature sites found by Gnanakaran, et al. Alternatively, it may be possible to identify functionally linked non-adjacent amino acids using correlation matrices to assess how disparate regions of the envelope protein vary in relation to each other, as has recently been done with HIV-1 Gag [45].

The present study shows that sequence variation at a specific locus within the envelope leader peptide facilitates virus transmission and/or propagation in a new host. The ability of amino acid shifts to mediate crucial transitions in viral ontogeny within the host has previously been observed with chemokine receptor tropism [46]: early viruses are almost exclusively CCR5-tropic and CXCR4 tropism arises later in infection. Just as evolution in viral cellular tropism may reflect changes in target cell availability, leader peptide evolution may reflect adaptation from a low viral load, target cell rich environment to a high virus load, target cell limited environment. We have modeled the role of viral infectivity in very early and in steady-state infection. Infectivity may be most important during the virus ramp up phase when sufficiently activated target cells are limited. We show, however, that at viral set-point, the ability of a variant to achieve numerical superiority through high reproductive rates appears to be more important than its ability to compete for a limited number of individual target cells via an enhanced infectivity.

But why might the position 12 signature be preferentially lost during chronic infection? Lowering envelope expression levels may be advantageous during chronic infection to escape anti-viral antibodies. Alternatively, different target cell populations may respond differently to changes in the signal peptide. The shift from CCR5 to CXCR4 tropism can potentially be explained by a shift in target cell populations as the virus expands into new niches. It is unclear if comparable cell type specificity in the position 12-determined phenotype plays a role late in infection, and whether the transmission phenotype may be lost, or become neutral. Studies of additional HIV-1 envelope signatures, their temporal and spatial association with the position 12 signature, and their biological effects will provide a more complete understanding of the selection pressures faced by the virus during acute and chronic infection.

## Supporting Information

Table S1 **Clinical characteristics of patient specimens.** Adapted from Keele, et al. Supplemental Tables, S1 – S4 [8], and Gnanakaran, et al, SuppS1. Fiebig classification of plasma specimens, as described previously [28]. Risk behavior: heterosexual (H) or men who have sex with men (MSM). Estimate to most recent common ancestor (MRCA) is an estimate of the time since infection, as determined by Keele, et al. [8]
(XLS)
